# Supplementary material for: Dimethyl Fumarate-Loaded Gellan Gum Hydrogels Can Reduce In Vitro Chemokine Expression in Oral Cells
Source: Int J Mol Sci. 2024 Aug 31;25(17):9485. doi: 10.3390/ijms25179485 (PMC11395421; doi:10.3390/ijms25179485)
Supplement: Supplementary file 1 [file ijms-25-09485-s001.zip › ijms-3113624-supplementary.pdf]

Supplement Figures

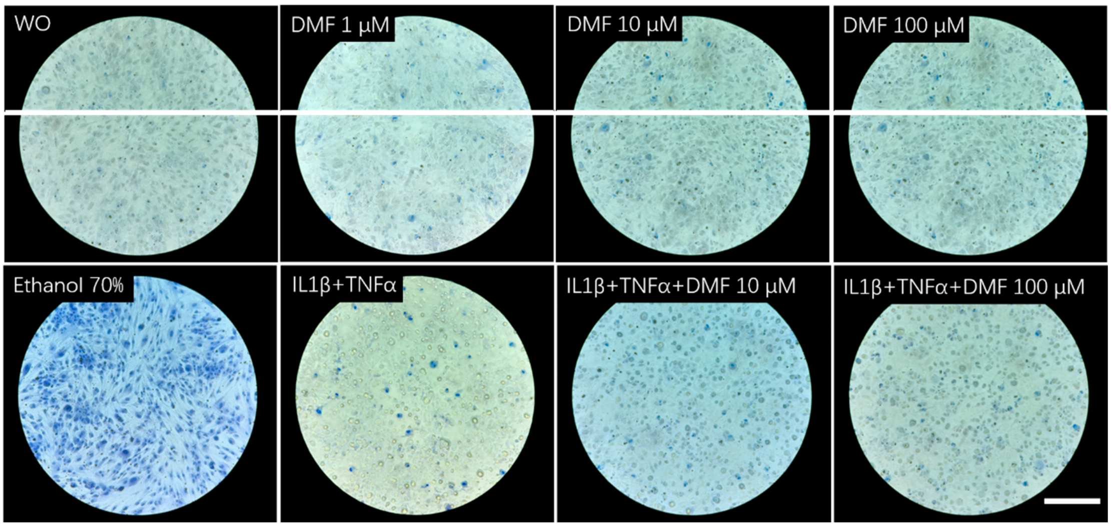

Supplement Figure S1. Representative images trypan blue staining of GF. Cells were exposed to the serum-free media with a serial concentration of DMF, with or without IL-1β and TNF-α, overnight and subjected to trypan blue staining assay. Scale bar represents 360 μm.

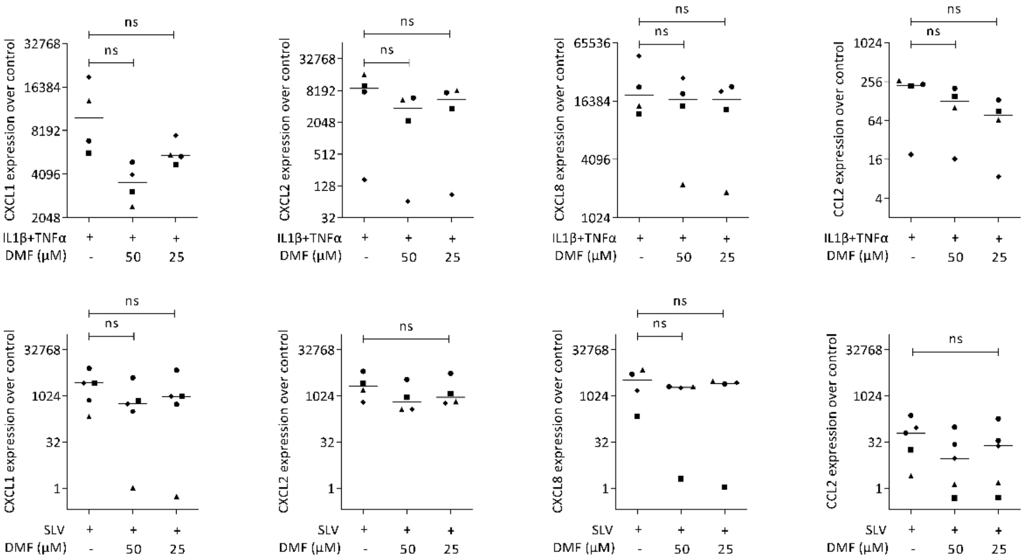

Supplement Figure S2. Gene expression of chemokine CXCL1, CXCL2, CXCL8, and CCL2 under DMF 50 μM, and 25 μM on IL-1β and TNF-α- and saliva (SLV)- induced gingival fibroblasts. ns stands for non-significant.

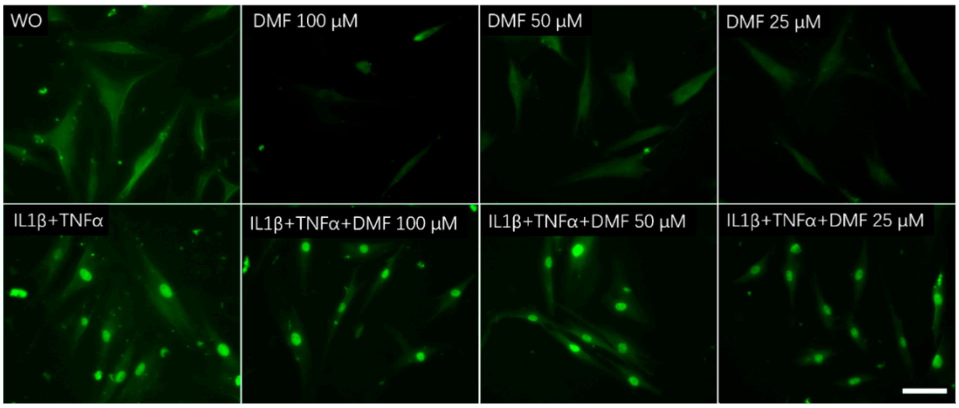

Supplement Figure S3. p65 nuclear translocation in GF cells. Both DMF and IL-1β+TNF-α induced the nuclear translocation of p65 indicated by the immunofluorescence staining. The scale bar represents 100 μm.

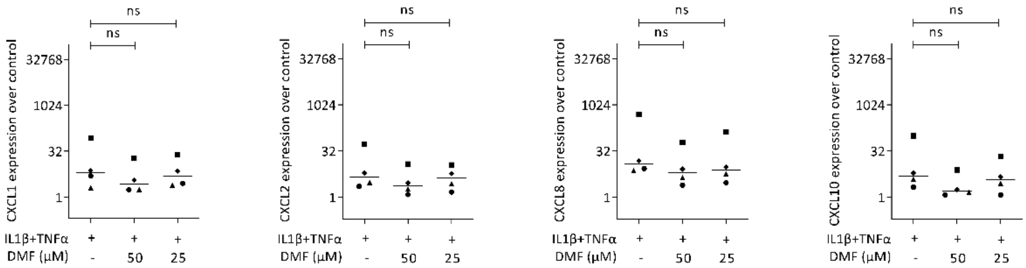

Supplement Figure S4. Gene expression of chemokine CXCL1, CXCL2, CXCL8, and CXCL10 under DMF 50 μM, and 25 μM on SLV-induced HSC2 cells. ns stands for non-significant.
